# Knowledge, Awareness, and Influence of the COVID-19 Pandemic on Students of Biomedical Faculties: A Cross-Sectional Study

**DOI:** 10.3390/dj13010028

**Published:** 2025-01-10

**Authors:** Bruno Špiljak, Luka Šimunović, Ana Marija Miličević, Marko Granić, Lana Bergman, Jasminka Peršec

**Affiliations:** 1 Department of Oral Medicine, University of Zagreb School of Dental Medicine, 10000 Zagreb, Croatia; bspiljak@sfzg.hr; 2 Department of Orthodontics, University of Zagreb School of Dental Medicine, 10000 Zagreb, Croatia; lsimunovic@sfzg.hr (L.Š.); amilicevic@sfzg.hr (A.M.M.); 3 Department of Oral Surgery, University of Zagreb School of Dental Medicine, 10000 Zagreb, Croatia; granic@sfzg.hr; 4 Department of Prosthodontics, University of Zagreb School of Dental Medicine, 10000 Zagreb, Croatia; 5 Clinical Department for Anesthesiology, Reanimatology and Intensive Care Medicine, University Hospital Dubrava, University of Zagreb School of Dental Medicine, 10000 Zagreb, Croatia; persec@sfzg.hr

**Keywords:** COVID-19, biomedical students, knowledge, media, psychological impact, vaccination, misinformation

## Abstract

**Background/Objectives**: The COVID-19 pandemic has had a profound impact on physical, mental, and social aspects of life worldwide. This study aimed to explore and compare differences in knowledge, awareness, behavior, and the psychological impact of the pandemic among students of biomedical faculties at the University of Zagreb. **Methods**: A cross-sectional study was conducted between 27 November 2020 and 19 January 2021 involving 518 students from the School of Dental Medicine, School of Medicine, Faculty of Pharmacy and Biochemistry, and Faculty of Veterinary Medicine. An anonymous online questionnaire was distributed, assessing participants’ knowledge about SARS-CoV-2, information sources, attitudes, and psychological responses. Data were analyzed using descriptive statistics and appropriate statistical tests. **Results**: The median knowledge score was 61.54%, with senior and female students demonstrating significantly more knowledge (*p* < 0.001 and *p* = 0.044, respectively). Students who consulted the scientific literature and official websites had higher knowledge levels (*p* < 0.001). Most participants used media and social networks for pandemic information, while scientific sources were underutilized. Psychological impacts were evident, with 46.3% expressing fear about the future and 25% reporting anxiety if they were to be infected. Additionally, those who engaged with the scientific literature were more likely to accept vaccination and showed lower levels of COVID-19 stigma. A majority (64.5%) believed that the media exaggerated the pandemic’s risks. **Conclusions**: Biomedical students demonstrated moderate knowledge about COVID-19, with a clear link between scientific literacy and more informed, less stigmatizing attitudes. This study underscores the importance of reliable information sources in shaping public health awareness and highlights the need for further education on COVID-19 symptoms and preventive measures.

## 1. Introduction

At the beginning of 2020, humanity faced an unprecedented threat to both physical and mental health in the form of the SARS-CoV-2 virus. The first case of infection was reported and confirmed in the Chinese city of Wuhan, the capital of Hubei province, on 31 December 2019 and was initially described as pneumonia of unknown etiology [1,2]. Soon after, the disease known as COVID-19, caused by the SARS-CoV-2 virus, spread to other parts of the world, prompting the World Health Organization (WHO) to declare a global pandemic on 11 March 2020 [3]. From the start of the pandemic to 23 June 2024, over 775 million cases of infection and more than 7 million deaths were recorded worldwide [4]. The typical clinical presentation of the disease includes respiratory symptoms such as coughing, shortness of breath, the temporary loss of smell and taste, and elevated body temperature. Severe symptoms include pneumonia and multiple organ failure caused by the virus, which significantly increase the risk of a fatal outcome [5,6].

In an effort to curb the spread of infection and reduce mortality, many countries introduced restrictions on daily life. For example, on 23 January 2020, the Chinese government suspended public transportation in Wuhan, followed by similar measures in many other Chinese cities [7]. This trend quickly spread globally, with governments implementing various restrictions, including social distancing, changes to or the cessation of work, movement limitations, and mandatory face masks [8,9]. Since these non-pharmacological measures did not prove highly effective, the need for pharmacological interventions, specifically a COVID-19 vaccine, arose.

The COVID-19 pandemic acted as a significant stressor for the general population, not only due to drastic lifestyle changes but also because of the virus’s unpredictability and the risks associated with infection. This, combined with an overwhelming amount of information—and fake news—led to increased anxiety and fear among the population [10]. In fact, fake news poses a serious public health issue. Lazar et al. [11] define fake news as false information that mimics legitimate media content in form but not in organizational process or intent. It overlaps with other types of informational disorders, such as “misinformation”, which is false or misleading information spread unintentionally, and “disinformation”, which refers to false information deliberately spread to deceive. The primary problem with misinformation is its rapid spread and deep-rooted presence within populations, driven largely by social media [12]. As a result, the WHO declared an “infodemic” alongside the viral pandemic, contributing to feelings of anxiety, fear, and uncertainty. Moreover, it has been shown that more people believe conspiracy theories and fake news than the official guidelines provided by authorities [13].

These extraordinary social conditions have led to an increased prevalence of anxiety and depressive disorders. Children and adolescents are particularly vulnerable, as they are more prone to developing neurotic disorders compared to adults [14]. With the onset of the pandemic, there was a noticeable rise in the incidence of psychological disorders among these age groups [15]. School and university closures, limited social interactions, the introduction of online classes, the loss of peer support, and isolation in their own homes significantly worsened the mental health of young people, especially university students [16,17]. Besides the aforementioned reasons, students are a particularly vulnerable group due to pre-existing concerns about the future, which were exacerbated by the pandemic [18].

As the number of cases and deaths increased, along with the rapid spread of misinformation, the need for role models who could positively influence society became apparent [19]. Healthcare workers and students in biomedical fields are especially qualified to promote health awareness and responsible health behavior [20]. Since they are a reliable source of medical information, they can influence their immediate environment by changing attitudes and perspectives regarding health-related issues [21].

The purpose of this study is to explore and compare differences in knowledge, awareness, behavior, and the psychological impact of the COVID-19 pandemic among students of biomedical faculties at the University of Zagreb, with an additional focus on dental students. Given their critical role in healthcare, an understanding of the unique challenges and responses of dental students during the pandemic is crucial. This study aims to address gaps in knowledge and awareness while evaluating the broader implications of students’ experiences on education and future professional responsibilities.

## 2. Materials and Methods

### 2.1. Study Design

The participants in this study were students from biomedical faculties at the University of Zagreb (School of Dental Medicine, School of Medicine, Faculty of Pharmacy and Biochemistry, and Faculty of Veterinary Medicine) during the period from 27 November 2020 to 19 January 2021. An online questionnaire created using Google Forms was sent to their email addresses. The questionnaire was completely anonymous, and participation in the study was voluntary. Since this was an online questionnaire, participants did not sign an informed consent form. Instead, they were required to read the informed consent text and confirm that they understood the purpose of and agreed to participate in the study, after which they were granted access to the questionnaire. The study was approved by the Ethics Committee of the School of Dental Medicine, University of Zagreb (number: 05-PA-30-XX-10/2020).

The questionnaire consisted of four sections (Appendix A). In the first section, general information about the participants was collected (gender, age, faculty attended, year of study), and the frequency of use for sources of information about the SARS-CoV-2 virus pandemic was assessed using a four-point scale (1—never used; 2—sometimes used; 3—often used; 4—always used). In the second section, knowledge about the SARS-CoV-2 virus and COVID-19 infection was registered. For this purpose, true/false questions were used, as well as questions with one or more correct answers. The knowledge score was calculated as the arithmetic mean of all 13 knowledge questions, where each question was scored as follows: a correct answer received a score of 1, and an incorrect answer received a score of 0.

The weight of each question was determined using the following formula:

$$Weight\;of\;the\;question=\frac{Variance\;of\;Question}{Sum\;of\;Variances\;of\;All\;Questions}$$

In the third section, using yes/no/don’t know questions, participants’ personal attitudes toward the COVID-19 pandemic were examined. Additionally, the question “How long do you think this pandemic will last?” was posed, offering multiple answer choices. In the fourth and final section, the psychological impact of the pandemic on participants’ lives was examined. Questions with the following answer choices were used: disagree/maybe/agree/don’t know. A question about coping mechanisms with the SARS-CoV-2 virus was also included, where participants could select one or more responses.

### 2.2. Validity and Reliability

The validity of the questionnaire used in this research was established through a multi-step process. Content validity was confirmed by consulting with an expert in the field, who reviewed the items for relevance and clarity. Construct validity was assessed by conducting a pilot study with a subset of participants (50), followed by exploratory factor analysis (EFA), which revealed a clear 4-factor structure, explaining 68% of the total variance. Cronbach’s alpha values ranged from 0.82 to 0.91 across the different scales, indicating high internal consistency.

### 2.3. Sample Size Analysis

A sample size analysis was conducted using an a priori power analysis for an F test (ANOVA: fixed effects, omnibus, one-way) to determine the required sample size. The analysis assumed an effect size of 0.25, a significance level of 0.05, and a desired power of 0.99, with 4 groups being compared. Based on these parameters, the total required sample size was 384 participants, or 96 participants per group, to achieve an actual power of 0.99. However, in the final study, we exceeded this minimum requirement and included 518 participants.

### 2.4. Statistical Analysis

Data were organized into tabular files using Microsoft Excel (Microsoft Inc., Redmond, Washington, DC, USA) and prepared for statistical analysis, which was conducted using Statistica software (TIBCO^®^ Statistica™ Version 13.5.0.17, Palo Alto, CA, USA). The statistical tests performed included the Chi-squared test and Cramér’s V test for association analysis, as well as the Kruskal–Wallis test with the post-hoc Dunn’s test for comparing knowledge levels.

## 3. Results

The study involved 518 students, of whom 26.6% were from the School of Dental Medicine, 23% were from the School of Medicine, 26.1% were from the Faculty of Veterinary Medicine, and 24.3% were from the Faculty of Pharmacy and Biochemistry. Women comprised 82.4% of the participants, while men accounted for 17.6%.

The median score for knowledge about SARS-CoV-2 was 61.54% (IQR 53.85–61.54), and there was no statistically significant difference between faculties. Third- to sixth-year students had statistically significantly more knowledge than first- and second-year students (*p* < 0.001), and women demonstrated more knowledge than men (*p* = 0.044), though the proportion of men and women in the study should be considered.

The highest percentage of correct responses was for questions about the virus’s origin (93.4%), and the lowest was for symptoms characteristic of COVID-19 (3.47%). Men had more knowledge about the virus’s origin (*p* = 0.018), while more women knew about virus transmission (*p* = 0.033). Regarding the nucleic acid component of the SARS-CoV-2 virus, there was a statistically significant difference in knowledge: 86.6% of medical students and 68.8% of dental students answered correctly (*p* = 0.001). The distribution of knowledge scores, broken down by students of different faculties, years of study, and genders, is shown in Table 1. The weight of each specific question toward the knowledge score is presented in Table 2.

**Figure 1:**
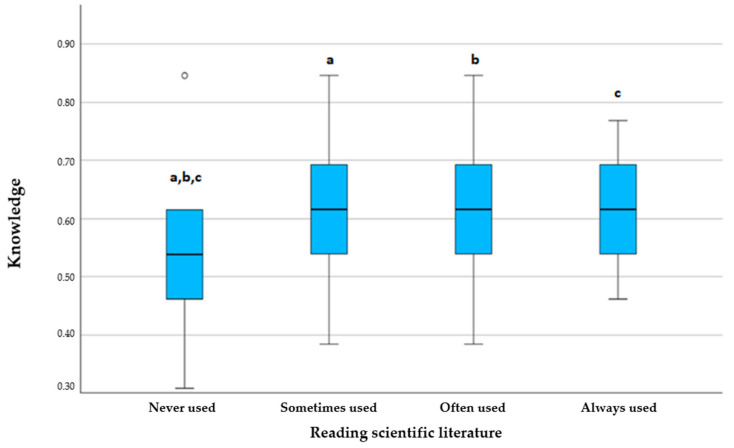
Comparison of knowledge scores based on the frequency of reading the scientific literature; a (*p* < 0.001), b (*p* = 0.011), c (*p* = 0.043). Kruskal–Wallis test with post-hoc Dunn’s test.

**Figure 2:**
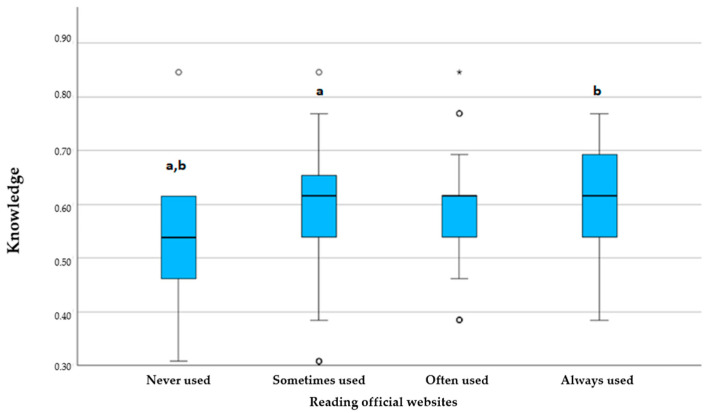
Comparison of knowledge scores based on the frequency of reading official websites; a (*p* < 0.0032), b (*p* = 0.015), * presents outlier result. Kruskal–Wallis test with post-hoc Dunn’s test.

The most commonly used sources of COVID-19 information were news and media (26.3%), followed by social media (24.3%) and friends and family members (24.3%), with the frequency of use not impacting the level of knowledge. The least used sources were official websites and the scientific literature, which were never utilized by 35.9% and 53.7% of respondents, respectively. However, reading the scientific literature significantly impacted knowledge, with those who read more showing greater knowledge (*p* < 0.001). A similar trend was observed among those who visited official websites (*p* = 0.038). There was a statistically significant difference between those who frequently used official websites and the scientific literature and those who used them less frequently (Figure 1 and Figure 2). Those who used better sources demonstrated more knowledge.

Responses to the questions “Do you think the COVID-19 pandemic will be successfully eradicated?” and “If I got infected, I would be afraid of how the healthcare system would take care of me” are shown in Table 3. The smallest proportion of pharmacy students (27.8%) believed that the pandemic would be successfully eradicated (*p* = 0.020). Moreover, 58.3% of all participants disagreed with the decisions of the National Civil Protection Headquarters of the Republic of Croatia. Interestingly, a higher proportion of dental (45.7%) and medical (47.1%) students compared to veterinary (30.3%) and pharmacy (25.4%) students agreed with the statement that they would be afraid of how the healthcare system would take care of them in case of COVID-19 infection (*p* = 0.008).

A quarter of respondents reported feelings of anxiety, discomfort, or fear if they tested positive for SARS-CoV-2. Additionally, 46.3% of all participants reported fear when thinking about the future. Furthermore, 60.6% of respondents who frequently watched the news believed it was necessary to inform the public daily about the number of new COVID-19 cases, compared to those who rarely (41.3%) or almost never (42.2%) watched the news (*p* = 0.027). Similarly, among participants who frequently read the scientific literature, 87.5% expressed willingness to get vaccinated, unlike those who rarely (52.1%) or almost never (41.3%) read it (*p* = 0.026). Those who would get vaccinated statistically demonstrated more knowledge than those who were unsure (*p* = 0.006). A similar trend was observed in responses to questions about the stigmatization of infected individuals (*p* = 0.017). The impact of media usage and attitudes on respondents’ opinions about the danger of the SARS-CoV-2 virus is shown in Table 4. A trend is displayed indicating that frequent information through news, official websites, and the scientific literature affects opinions about the perceived danger of the virus. Additionally, attitudes toward vaccination, the need for daily public reporting on new cases, and the stigmatization of those affected are greatly dependent on the opinion about the virus’s danger.

**Figure 3:**
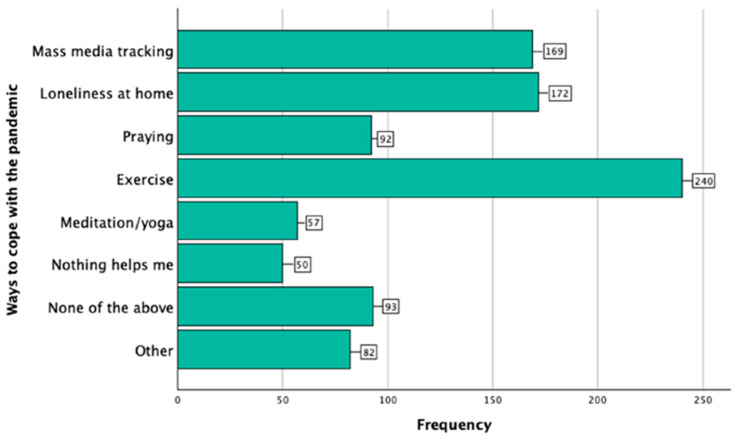
Distribution of responses to the multiple-choice question regarding mechanisms participants use to cope with the pandemic.

The most commonly reported ways to cope with the pandemic are shown in Figure 3.

## 4. Discussion

The COVID-19 pandemic was a significant event that impacted the daily lives of individuals, especially students from biomedical faculties—future experts in clinical and basic sciences. This study compared four biomedical faculties in Zagreb—the School of Dental Medicine, School of Medicine, Faculty of Pharmacy and Biochemistry, and Faculty of Veterinary Medicine—during the uncertain period of 2021.

The results of the current study show that the median knowledge score of all respondents was 61.54%, with no significant differences between faculties. Similar knowledge levels were recorded in Turkey, Saudi Arabia, Egypt, India, and Nepal, while higher levels were noted in Serbia, Ethiopia, Iran, and China [22,23,24,25,26,27,28,29,30,31,32]. These differences may stem from variations in information sources, faculty strategies, and assessment methods.

No significant differences in knowledge between biomedical faculties were observed, indicating similar information sources used during the pandemic. These results align with findings from Nepal [33], suggesting that dental students, despite their focus on oral health, possess comparable knowledge about broader public health crises, emphasizing the integration of such topics into dental curricula. This underscores the value of integrating pandemic education into all biomedical curricula, as noted in previous studies [29,34,35,36].

Senior-year students exhibited more knowledge than junior-year students, likely due to their greater clinical exposure. This trend has been observed in numerous studies, emphasizing the impact of education level and clinical experience on pandemic knowledge [29,35,37,38,39,40,41,42]. Female students also demonstrated better knowledge than males, consistent with prior research, although exceptions exist [19,29,35,43,44,45].

Questions related to the virus’s origin were answered most correctly, while those related to symptoms showed gaps in knowledge, highlighting areas for targeted education (Table 2). Women showed a greater understanding of virus transmission, a finding supported by several studies [27,44,46]. Dental students demonstrated slightly lower awareness about SARS-CoV-2’s origin compared to medical students, potentially reflecting the reduced focus on their preclinical courses compared to medical students, suggesting the need for interdisciplinary education to address knowledge gaps [47]. Studies indicate that dental students’ knowledge about COVID-19, including its transmission and preventive measures, is significantly related to their educational level and the type of institution they attend [48,49]. Furthermore, dental students often face challenges in accessing comprehensive training on infectious diseases within their curriculum, which can lead to the aforementioned knowledge gaps [50,51].

Additionally, more than half of the respondents in the study by Gohel et al. [21] were unaware of correct information regarding the virus’s origin, highlighting knowledge gaps in certain disease aspects. Medical students demonstrated better knowledge of SARS-CoV-2’s nucleic acid composition compared to dental students, likely due to their more extensive coursework in microbiology and infectious diseases. To address these discrepancies, it is essential for dental education programs to enhance their curricula by integrating more comprehensive training in infectious diseases and public health issues, ensuring that dental students are equally prepared to manage risks associated with pandemics like COVID-19.

While some students demonstrated good knowledge of symptoms, the majority, as noted by Jha et al. [33], lacked awareness of all characteristic symptoms, emphasizing the need for targeted education. A study conducted by Khasawneh et al. [52] suggests that providing accurate information through mass media could enhance awareness and knowledge. Our findings also show that students using credible sources, such as the scientific literature, had significantly better knowledge compared to those relying on social media, consistent with previous research [44,52,53] (Figure 1 and Figure 2).

In terms of information sources, the most frequently used by respondents were social media and family and friends, while the least utilized sources were the scientific literature and official websites. This trend was particularly noticeable among dental students, who may benefit from initiatives promoting the use of evidence-based resources, such as scientific journals and official health organization websites. The distribution of responses to the question “Do you think the SARS-CoV-2 virus is dangerous?” regarding the impact of the media used and attitudes on respondents’ opinions about the danger of the SARS-CoV-2 virus among different faculties is presented in the Supplementary Materials Section (Table S1). These results align with other studies, showing that social media use dominates among younger generations but carries the risk of misinformation [3,4,11,34,49,54,55,56,57,58,59,60,61,62,63]. To address this, the WHO declared an “infodemic” alongside the pandemic to highlight the dangers of misinformation [3,11].

Differences in source reliance may reflect varying public health investments across countries. Some countries prioritize official websites like the Ministry of Health or the WHO, making them more accessible within academic communities [21,37,39,40,64,65,66]. Pharmacy students showed the lowest optimism about eradicating the pandemic, likely due to their understanding of viral behavior and challenges in long-term suppression (Table 3). Additionally, 58.3% of all respondents expressed distrust in Croatia’s governmental measures, mirroring trends in Ecuador but contrasting with higher trust levels in the United Arab Emirates (UAE) and Serbia, where stricter measures likely boosted confidence [29,67,68]. Dental and medical students expressed greater concern about healthcare system readiness (Table 3). This heightened anxiety stems from their direct exposure to clinical environments and firsthand observation of resource strain. Kim et al. [69] and Sarikaya [70] reported similar findings, showing that clinical students experience heightened anxiety regarding their health and the healthcare system’s preparedness. Singh et al. [71] further emphasized these concerns, linking them to fears of inadequate personal protective equipment (PPE) and overwhelmed systems. Such concerns reflect broader distrust in state institutions and hospital systems operating under strict pandemic protocols. Similar trends of fear and anxiety among biomedical students during health crises have been widely reported [52,72].

During the pandemic, students generally experienced anxiety and fear, which is typical during global health crises. Our study revealed heightened levels of anxiety among students, often attributed to their poorer mental health compared to the general population [72,73]. Academic stress and temperament differences further contributed to these feelings [74,75]. Concerns about personal and family health were key anxiety drivers, aligning with prior research [68,76,77]. Similarly to findings in Jordan, Turkey, and the UAE, anxiety about the healthcare system’s capacity to manage infections was prevalent, reflecting broader institutional distrust [48,68,70].

Respondents who followed pandemic news frequently highlighted the importance of daily updates (Table 4). While credible news sources informed public perception, misinformation from social media amplified anxiety and confusion, as noted by Lazer et al. [11]. Studies emphasize the dual role of the media in disseminating accurate information while mitigating public fears [78,79].

Students engaging with the scientific literature showed greater vaccine acceptance and lower stigma levels (Table 4). Scientific literacy fosters proactive health behaviors, though cultural and personal beliefs may also shape vaccine decisions [80,81,82]. Persistent efforts to reduce stigma through education and accurate health policies remain essential [63,71,83]. Dental students, in particular, demonstrated heightened concern about the stigma associated with their role as healthcare providers during the pandemic. This emphasizes the need for targeted support to address the unique challenges faced by dental students in balancing public perception and professional responsibilities.

Conspiracy theories and misinformation continue to influence perceptions, with Mohmmed et al. [84] noting widespread belief in synthetic virus origins among participants. As the WHO declared, combating the “infodemic” requires reliable information sources to counter false narratives and enhance public understanding [85].

Similarly to in Al-Ghazali et al. [59], 63.7% of respondents considered COVID-19 dangerous, though 62.2% felt that media exaggerated its risks, potentially reducing adherence to safety measures. Public education must balance risk communication with clarity to combat misinformation’s effects [86].

Behavioral changes during the pandemic included reduced self-care and a reliance on coping mechanisms like exercise and media updates (Figure 3). These findings align with Savitsky et al. [73] and Cao et al. [87], highlighting the pandemic’s psychological toll on students. Ochnik et al. [88] reported that students often resorted to coping mechanisms such as physical activity and withdrawing into isolation, while media consumption played a dual role, sometimes providing necessary information but also contributing to anxiety.

It is important to consider the limitations of this study. One of the main issues is the possibility of response bias, where participants may provide socially desirable answers or fail to accurately reflect their true opinions and experiences. Additionally, this type of study (cross-sectional) only provides a snapshot of the current state at a specific moment in time, without insight into causal relationships or changes over time. Furthermore, survey questionnaires often rely on participants’ self-assessment, which can lead to inaccuracies due to subjectivity or misinterpretation of the questions.

## 5. Conclusions

This study demonstrates that biomedical students at the University of Zagreb had a moderate level of knowledge about COVID-19, with senior and female students demonstrating better understanding. While most students were aware of the virus’s origin, awareness of symptoms was lacking, emphasizing the need for more targeted education. Students who relied on scientific sources had significantly higher knowledge, while social media use did not improve understanding. Psychological impact was evident, with many students expressing fear and anxiety, though those engaged with the scientific literature were more likely to accept vaccination and showed less stigmatization. These findings emphasize the crucial role of healthcare students as informed advocates for public health, particularly in their future roles in educating the broader population. Efforts to combat misinformation and promote scientific literacy should remain a priority in mitigating the impacts of future public health crises.

## Figures and Tables

**Table 1:** The distribution of knowledge scores across different faculties, years of study, and genders.

Faculty	Year of Study	Gender	Median	Percentile 25	Percentile 75	Faculty	Year of Study	Gender	Median	Percentile 25	Percentile 75
Dental medicine	1st	Male	0.46	0.38	0.69	Veterinary	1st ^a^	Male	0.50	0.46	0.62
		Female	0.54	0.46	0.62			Female	0.46	0.46	0.62
		Total	0.54	0.46	0.62			Total	0.46	0.46	0.62
	2nd	Male ^+^	0.46	0.46	0.46		2nd	Male	0.54	0.54	0.62
		Female ^+^	0.62	0.54	0.62			Female	0.54	0.46	0.62
		Total	0.54	0.46	0.62			Total	0.54	0.46	0.62
	3rd	Male	0.54	0.54	0.54		3rd ^a^	Male	0.54	0.54	0.62
		Female	0.62	0.54	0.69			Female	0.62	0.54	0.69
		Total	0.62	0.54	0.69			Total	0.62	0.54	0.69
	4th	Male	0.69	0.62	0.73		4th	Male	0.65	0.62	0.73
		Female	0.62	0.54	0.62			Female	0.62	0.54	0.62
		Total	0.62	0.54	0.69			Total	0.62	0.54	0.62
	5th	Male	0.46	0.46	0.54		5th	Male	0.58	0.50	0.65
		Female	0.54	0.46	0.69			Female	0.58	0.54	0.62
		Total	0.54	0.46	0.69			Total	0.58	0.54	0.62
	6th	Male	0.54	0.54	0.54		6th	Male	0.00	0.00	0.00
		Female	0.54	0.46	0.65			Female	0.62	0.62	0.62
		Total	0.54	0.46	0.62			Total	0.62	0.62	0.62
	Total		0.54	0.46	0.62		Total		0.62	0.54	0.69
Medicine	1st	Male	0.54	0.54	0.54	Pharmacy	1st	Male	0.54	0.38	0.69
		Female	0.62	0.46	0.62			Female	0.54	0.46	0.62
		Total	0.58	0.46	0.62			Total	0.54	0.46	0.62
	2nd	Male	0.62	0.54	0.69		2nd ^b,c,d^	Male	0.46	0.42	0.54
		Female	0.54	0.54	0.69			Female	0.46	0.38	0.54
		Total	0.58	0.54	0.69			Total	0.46	0.38	0.54
	3rd	Male	0.62	0.58	0.65		3rd	Male	0.62	0.58	0.62
		Female	0.62	0.54	0.69			Female	0.54	0.46	0.62
		Total	0.62	0.54	0.69			Total	0.62	0.46	0.62
	4th	Male	0.54	0.54	0.54		4th ^b^	Male	0.62	0.54	0.69
		Female	0.62	0.54	0.69			Female	0.62	0.54	0.69
		Total	0.62	0.54	0.69			Total	0.62	0.54	0.69
	5th	Male	0.62	0.54	0.62		5th ^c^	Male ^++^	0.46	0.46	0.46
		Female	0.62	0.54	0.69			Female ^++^	0.62	0.54	0.69
		Total	0.62	0.54	0.69			Total	0.62	0.54	0.69
	6th	Male	0.69	0.54	0.69		6th ^d^	Male	0.54	0.46	0.62
		Female	0.62	0.46	0.69			Female	0.62	0.54	0.65
		Total	0.65	0.54	0.69			Total	0.62	0.54	0.62
	Total		0.62	0.54	0.69		Total		0.58	0.46	0.62

Signs (^+^ and ^++^) and letters (^a,b,c,d^) indicate statistically significant differences between genders and years of study; ^+^ (*p* = 0.019), ^++^ (*p* = 0.030), ^a^ (*p* = 0.048), ^b^ (*p* = 0.03), ^c^ (*p* < 0.001), ^d^ (*p* = 0.019).

**Table 2:** Descriptive statistics for individual questions, including their weights in the overall knowledge score calculation.

Questions	N	Mean	Standard Deviation	Variance	Weight
Which statement about coronaviruses is correct:	518	0.82	0.381	0.145	0.093
2. SARS-CoV-2. the virus that causes the disease COVID-19 is a DNA virus?	518	0.80	0.400	0.160	0.102
3. The origin of the SARS-CoV-2 virus is believed to be:	518	0.93	0.248	0.061	0.039
4. What is the incubation period of SARS-CoV-2?	518	0.65	0.478	0.228	0.146
5. Of the following. all are possible transmission routes of SARS-CoV-2 except:	518	0.05	0.222	0.050	0.032
6. What is considered “close contact”?	518	0.87	0.338	0.114	0.073
7. From the listed symptoms. mark those that are not characteristic of COVID-19:	518	0.03	0.183	0.034	0.028
8. Of the listed. mark all complications of the COVID-19 disease:	518	0.05	0.226	0.051	0.033
9. Of the listed. mark the groups of at-risk patients. i.e., those more likely to develop complications from COVID-19:	518	0.80	0.400	0.160	0.102
10. What is the treatment for a COVID-19 infection?	518	0.32	0.467	0.218	0.139
11. The SARS-CoV-2 virus can survive on objects for several hours	518	0.93	0.255	0.065	0.042
12. Coagulation dysfunction is one of the main causes of death in patients with COVID-19	518	0.28	0.451	0.204	0.130
13. People suffering from a COVID-19 infection cannot transmit the virus if they do not have a fever	518	0.92	0.276	0.076	0.049

**Table 3:** Distribution of responses to the questions “Do you think the COVID-19 pandemic will be successfully eradicated?” and “If I got infected, I would be afraid of how the healthcare system would take care of me”.

Questions	Answer	Dental Medicine N (%)	Medicine N (%)	Veterinary N (%)	Pharmacy N (%)	*p* Value
Do you think the COVID-19 pandemic will be successfully eradicated?	Yes	66 (47.8)	51 (42.9)	55 (40.7)	35 (27.8)	χ^2^ = 14.982 df = 6 *p* = 0.020
	No	41 (29.7)	28 (23.5)	39 (28.9)	44 (34.9)	
	Don’t know	31 (22.5)	40 (33.6)	41 (30.4)	47 (37.3)	
If I got infected. I would be afraid of how the health care system would take care of me	I agree	63 (45.7)	56 (47.1)	45 (33.3)	32 (25.4)	χ^2^ = 22.214 df = 9 *p* = 0.008
	Maybe	37 (26.8)	36 (30.3)	47 (34.8)	44 (34.9)	
	I do not agree	30 (21.7)	25 (21.0)	38 (28.1)	45 (35.7)	
	Don’t know	8 (5.8)	2 (1.7)	5 (3.7)	5 (4.0)	

**Table 4:** Distribution of responses to the question “Do you think the SARS-CoV-2 virus is dangerous?” regarding the impact of the media used and attitudes on respondents’ opinions about the danger of the SARS-CoV-2 virus.

Frequency of Use/Response	Yes N (%)	No N (%)	Don’t Know N (%)	*p* Value
News and media				
Never used	33 (51.6)	16 (25.0)	12 (23.4)	0.004
Sometimes used	103 (56.0)	43 (23.4)	38 (20.7)	
Often used	114 (68.7)	24 (14.5)	28 (16.9)	
Always used	80 (76.9)	13 (12.5)	11 (10.6)	
Official websites				
Never used	109 (58.6)	48 (25.8)	29 (15.6)	0.011
Sometimes used	119 (62.0)	32 (16.7)	41 (21.4)	
Often used	60 (69.0)	13 (14.9)	14 (16.1)	
Always used	42 (79.2)	3 (5.7)	8 (15.1)	
Scientific literature				
Never used	162 (58.3)	63 (22.7)	53 (19.1)	0.025
Sometimes used	109 (66.1)	26 (15.8)	30 (18.2)	
Often used	45 (76.3)	5 (8.5)	9 (15.3)	
Always used	14 (87.5)	2 (12.5)	0 (0.0)	
“If the vaccine is available I would take it.”				
Yes	200 (77.5)	28 (10.9)	30 (11.6)	<0.001
No	41 (33.6)	48 (39.3)	33 (27.0)	
Don’t know	89 (64.5)	20 (14.5)	29 (21.0)	
“I believe that it is important to inform the public daily about the number of new cases of COVID-19 infection.”				
Yes	202 (80.8)	20 (8.0)	28 (11.2)	<0.001
No	90 (41.5)	72 (33.2)	55 (25.3)	
Don’t know	38 (74.5)	4 (7.8)	9 (17.6)	
“I believe that people suffering from the infection of COVID-19 are stigmatized.”				
Yes	140 (71.1)	33 (16.8)	24 (12.2)	0.007
No	144 (57.1)	56 (22.2)	52 (20.6)	
Don’t know	46 (66.7)	7 (10.1)	16 (23.2)	

## Data Availability

Data are available upon request to the corresponding author.

## Supplementary Materials

The following supporting information can be downloaded at https://www.mdpi.com/article/10.3390/dj13010028/s1, Table S1: Distribution of responses to the question “Do you think the SARS-CoV-2 virus is dangerous?” regarding the impact of the media used and attitudes on respondents’ opinions about the danger of the SARS-CoV-2 virus among different faculties.

## Appendix A

The survey used in the present study (* indicates correct answer(s)):

Sociodemographic characteristics

I. Select your gender: Male, Female
II. Select your year of birth

1995, 1996, 1997

1998, 1999

2000, 2001, 2002

before 1995

III. Select the faculty you are attending

School of Dental Medicine University of Zagreb

School of Medicine University of Zagreb

Faculty of Pharmacy and Biochemistry University of Zagreb

Faculty of Veterinary Medicine University of Zagreb

IV. Select your year of study

1 year

2 year

3 year

4 year

5 year

6 year

postgraduate study

V. How would you rate the frequency of your use of the listed sources of information about the SARS-CoV-2 virus pandemic as a global threat, according to the scale provided?
  (a) News, media (TV, radio, newspapers, etc.)

Never used

Sometimes used

Often used

Always used

(b) Social media (e.g., Facebook, Twitter, YouTube, Instagram…)

Never used

Sometimes used

Often used

Always used

(c) Official websites of the Government and the Croatian Institute of Public Health, as well as other public health websites (e.g., WHO, www.koronavirus.hr.)

Never used

Sometimes used

Often used

Always used

(d) Academic research papers published in scientific journals and accessible through virtual databases on bioscience and biomedical topics (e.g., PubMed...)

Never used

Sometimes used

Often used

Always used

(e) A family member, colleague, or friend

Never used

Sometimes used

Often used

Always used

⁠

Knowledge about COVID-19

(1) Which statement about coronaviruses is correct:
  (a) Coronaviruses are viruses that circulate among animals. All viruses of this group can transfer to humans. Once they transfer from animals to humans, they can spread among humans.
  (b) Coronaviruses are viruses that circulate among animals, but some of them can transfer to humans. Once they transfer from animals to humans, they can spread among humans. *
  (c) Coronaviruses are viruses that circulate among humans, but some of them can transfer to animals. Once they transfer from humans to animals, they can spread among animals.
  (d) Coronaviruses are viruses that circulate among humans. All viruses of this group can transfer to animals. Once they transfer from humans to animals, they can spread among animals.
(2) Is SARS-CoV-2, the virus responsible for COVID-19, a DNA virus?

True, False*

(3) The origin of the SARS-CoV-2 virus is believed to be:

Bats*

Snakes

Fish

Camels

Rodents

(4) What is the incubation period of SARS-CoV-2?

2–7 days

2–14 days*

7–14 days

7–21 days

None of the above

(5) Of the following, all are possible transmission routes of SARS-CoV-2 except (Select all that apply):

Air

Direct contact

Fecal-oral route

Saliva and respiratory droplets from an infected person

Sexual intercourse*

Kissing and handshakes

Using items of an infected person

Consumption of certain foods*

(6) What is considered „close contact“?
  (a) A person at least 3 m away from a person infected with COVID-19 for a prolonged period
  (b) Face-to-face contact, less than 2 m from a person infected with COVID-19 for more than 15 min
  (c) Direct contact with secretions of an infected person (cough, serum, blood, etc.)
  (d) B + C*
  (e) A + C

(7) Which of the following symptoms are not characteristic of COVID-19 (Select all that apply)?

Fever

Headache

Blurred vision*

Muscle pain (myalgia)

Runny nose

Sore throat

Sneezing*

Diarrhea and vomiting

Dry cough

Productive cough*

Shortness of breath

Loss of smell

Loss of taste

Skin changes (e.g., rash)*

(8) Which of the following are complications of COVID-19 (Select all that apply)?

Neuropathy

Bronchitis*

Multiple organ failure*

Pneumonia*

Coagulation dysfunction*

Hyperglycemia

Diseases causing respiratory collapse*

(9) Risk groups for COVID-19 complications include (Select all that apply):

Adults

People with chronic diseases (e.g., diabetes, cancer, etc.)*

Immunocompromised individuals*

Older adults*

Children under 5 years old

(10) What is the therapy for COVID-19 infection?

Supportive therapy*

Antiviral therapy

Vaccination

A + B

None of the above

(11) The SARS-CoV-2 virus can survive on objects for several hours.

True*, False

(12) Coagulation dysfunction is one of the main causes of death in COVID-19 patients.

True*, False

(13) People infected with COVID-19 cannot transmit the virus if they do not have a fever.

True, False*

⁠

Attitudes about COVID-19

(1) How long do you think this pandemic will last?

1 month

3 months

6 months

12 months

More than 1 year

(2) Do you think the SARS-CoV-2 virus is dangerous?

Yes

No

I don’t know

(3) Do you think the COVID-19 pandemic will be successfully eradicated?

Yes

No

I don’t know

(4) Do you agree with the decisions made by the National Civil Protection Headquarters of the Republic of Croatia?

Yes

No

I don’t know

(5) Do you consider the COVID-19 pandemic part of a global conspiracy theory?

Yes

No

I don’t know

(6) Do you think the infection can be treated at home without medical intervention?

Yes

No

I don’t know

(7) I believe that frequent hand washing with soap or alcohol-based hand sanitizers will reduce the likelihood of COVID-19 infection.

Yes

No

I don’t know

(8) I believe that wearing a mask will reduce the likelihood of COVID-19 infection.

Yes

No

I don’t know

(9) I would agree to go into quarantine if I had contact with someone who has COVID-19.

Yes

No

I don’t know

(10) If a vaccine against SARS-CoV-2 was available, I would get vaccinated.

Yes

No

I don’t know

(11) I believe it is important to be regularly informed about the number of new COVID-19 cases.

Yes

No

I don’t know

(12) I believe it is important to be informed about the newly adopted preventive measures by the National Civil Protection Headquarters of the Republic of Croatia.

Yes

No

I don’t know

(13) I believe that people infected with COVID-19 are stigmatized.

Yes

No

I don’t know

⁠

Psychological impact of the COVID-19 pandemic

(1) From the options provided, choose your coping mechanisms with the SARS-CoV-2 virus:

Mass media tracking

Loneliness at home

Praying

Exercise

Meditation/yoga

Nothing helps me

None of the above

(2) If a family member gets infected with COVID-19, I would want it to remain a secret.

Agree

Maybe

Disagree

I don’t know

(3) If I got infected, I would be afraid of how the healthcare system would care for me.

Agree

Maybe

Disagree

I don’t know

(4) If I were potentially infected, I wouldn’t get tested just to avoid self-isolation.

Agree

Maybe

Disagree

I don’t know

(5) If I were infected, I would inform my close contacts about the potential risk.

Agree

Maybe

Disagree

I don’t know

(6) If I tested positive for SARS-CoV-2, I would feel anxious, uncomfortable, or scared.

Agree

Maybe

Disagree

I don’t know

(7) I feel responsible for the health of my household members, so I follow preventive measures.

Agree

Maybe

Disagree

I don’t know

(8) I believe the media have a negative impact on the mental health of the general population.

Agree

Maybe

Disagree

I don’t know

(9) I avoid talking about COVID-19 infection.

Agree

Maybe

Disagree

I don’t know

(10) I am afraid to think about the future.

Agree

Maybe

Disagree

I don’t know

(11) I have lost motivation to carry out daily tasks and obligations.

Agree

Maybe

Disagree

I don’t know

(12) I have stopped caring about my appearance.

Agree

Maybe

Disagree

I don’t know

(13) During the pandemic, I have become more prone to addictions (alcohol, cigarettes, unhealthy food, etc.).

Agree

Maybe

Disagree

I don’t know

(14) I feel like I have no control over my life.

Agree

Maybe

Disagree

I don’t know

(15) I am tenser and more nervous than usual.

Agree

Maybe

Disagree

I don’t know

(16) I have an inexplicable fear of catastrophic events.

Agree

Maybe

Disagree

I don’t know

(17) Recently, I have experienced sudden panic attacks.

Agree

Maybe

Disagree

I don’t know

(18) My sleep pattern has remained unchanged.

Agree

Maybe

Disagree

I don’t know

(19) I can focus/concentrate when necessary.

Agree

Maybe

Disagree

I don’t know

(20) I can still laugh and find positivity in the world around me.

Agree

Maybe

Disagree

I don’t know

(21) Despite the negatively changed reality, I try to stay optimistic.

Agree

Maybe

Disagree

I don’t know

(22) I try to share my emotions with others.

Agree

Maybe

Disagree

I don’t know

(23) I cannot accept the situation we are currently in.

Agree

Maybe

Disagree

I don’t know
