# Supplementary material for: Knowledge, Awareness, and Influence of the COVID-19 Pandemic on Students of Biomedical Faculties: A Cross-Sectional Study
Source: Dent J (Basel). 2025 Jan 10;13(1):28. doi: 10.3390/dj13010028 (PMC11763484; doi:10.3390/dj13010028)
Supplement: Supplementary file 1 [file dentistry-13-00028-s001.zip › dentistry-3251454-supplementary.pdf]

Table S1: Distribution of responses to the question "Do you think the SARS-COV-2 virus is dangerous?" regarding the impact of the media used and attitudes on respondents' opinions about the danger of the SARS-CoV-2 virus among different faculties.

**Faculty**	**Type of media/attitudes**	**Frequency of use**	**Yes N (%)**	**No N (%)**	**I don't know N (%)**	**P value**
Dental medicine	News and media	Never used	6 (50.0)	3 (25.0)	3 (25.0)	
		Sometimes used	30 (57.7)	14 (26.9)	8 (15.4)	
		Often used	27 (61.4)	11 (25.0)	6 (13.6)	
		Always used	22 (73.3)	3 (10.0)	5 (16.7)	
	Official websites	Never used	31 (56.4)	15 (27.3)	9 (16.4)	
		Sometimes used	29 (63.0)	9 (19.6)	8 (17.4)	
		Often used	18 (66.7)	6 (22.2)	3 (11.1)	
		Always used	7 (70.0)	1 (10.0)	2 (20.0)	
	Scientific literature	Never used	51 (58.0)	23 (26.1)	14 (15.9)	
		Sometimes used	22 (59.5)	7 (18.9)	8 (21.6)	
		Often used	10 (90.9)	1 (9.1)	0 (0.0)	
		Always used	2 (100.0)	0 (0.0)	0 (0.0)	
	"If the vaccine is available, I would take it."	Yes	53 (81.5)	8 (12.3)	4 (6.2)	<0.001
		No	11 (35.5)	11 (35.5)	9 (29.0)	
		Don't know	21 (50.0)	12 (28.6)	9 (21.4)	
	"I believe that it is important to inform	Yes	58 (79.5)	10 (13.7)	5 (6.8)	<0.001
		No	20 (35.7)	20 (35.7)	16 (28.6)	

	the public daily about the number of new cases of COVID-19 infection."	Don't know	7 (77.8)	1 (11.1)	1 (11.1)	
	"I believe that people suffering from the infection of COVID-19 are stigmatized."	Yes	32 (71.1)	10 (22.2)	3 (6.7)	
		No	43 (54.4)	19 (24.1)	17 (21.5)	
		Don't know	10 (71.4)	2 (14.3)	2 (14.3)	
Medicine	News and media	Never used	8 (53.3)	3 (20.0)	4 (26.7)	
		Sometimes used	19 (54.3)	9 (25.7)	7 (20.0)	
		Often used	39 (83.0)	3 (6.4)	5 (10.6)	
		Always used	17 (77.3)	3 (13.6)	2 (9.1)	
	Official websites	Never used	20 (57.1)	9 (25.7)	6 (17.1)	
		Sometimes used	36 (72.0)	7 (14.0)	7 (14.0)	
		Often used	14 (77.8)	2 (11.1)	2 (11.1)	
		Always used	13 (81.3)	0 (0.0)	3 (18.8)	
	Scientific literature	Never used	31 (58.5)	12 (22.6)	10 (18.9)	
		Sometimes used	34 (75.6)	6 (13.3)	5 (11.1)	
		Often used	13 (81.3)	0 (0.0)	3 (18.8)	
		Always used	5 (100.0)	0 (0.0)	0 (0.0)	
	"If the vaccine is available, I would take it."	Yes	44 (80.0)	5 (9.1)	6 (10.9)	
		No	9 (32.1)	10 (35.7)	9 (32.1)	<0.001
		Don't know	30 (83.3)	3 (8.3)	3 (8.3)	
	"I believe that it is important to inform the public daily about the number of new cases of COVID-19 infection."	Yes	48 (87.3)	4 (7.3)	3 (5.5)	
		No	22 (45.8)	12 (25.0)	14 (29.2)	<0.001
		Don't know	13 (81.3)	2 (12.5)	1 (6.3)	
	"I believe that people suffering from the infection of COVID-19 are stigmatized."	Yes	44 (77.2)	7 (12.3)	6 (10.5)	
		No	27 (56.3)	10 (20.8)	11 (22.9)	
		Don't know	12 (85.7)	1 (7.1)	1 (7.1)	
Veterinary	News and media	Never used	9 (56.3)	5 (31.3)	2 (12.5)	
		Sometimes used	32 (60.4)	10 (18.9)	11 (20.8)	
		Often used	21 (53.8)	7 (17.9)	11 (28.2)	
		Always used	22 (81.5)	4 (14.8)	1 (3.7)	
	Official websites	Never used	29 (58.0)	14 (28.0)	7 (14.0)	
		Sometimes used	32 (61.5)	8 (15.4)	12 (23.1)	
		Often used	10 (55.6)	3 (16.7)	5 (27.8)	
		Always used	13 (86.7)	1 (6.7)	1 (6.7)	
	Scientific literature	Never used	36 (56.3)	12 (18.8)	16 (25.0)	
		Sometimes used	31 (66.0)	10 (21.3)	6 (12.8)	
		Often used	13 (72.2)	2 (11.1)	3 (16.7)	
		Always used	4 (66.7)	2 (33.3)	0 (0.0)	
	"If the vaccine is available, I would take it."	Yes	63 (72.4)	10 (11.5)	14 (16.1)	
		No	6 (26.1)	13 (56.5)	4 (17.4)	<0.001
		Don't know	15 (60.0)	3 (12.0)	7 (28.0)	
	"I believe that it is important to inform the public daily about the number of new cases of COVID-19 infection."	Yes	47 (85.5)	2 (3.6)	6 (10.9)	
		No	26 (40.6)	24 (37.5)	14 (21.9)	<0.001
		Don't know	11 (68.8)	0 (0.0)	5 (31.3)	
	"I believe that people	Yes	34 (65.4)	11 (21.2)	7 (13.5)	<0.001

	suffering from the infection of COVID-19 are stigmatized.”	No	38 (61.3)	13 (21.0)	11 (17.7)	
		Don't know	12 (57.1)	2 (9.5)	7 (33.3)	
Pharmacy	News and media	Never used	10 (47.6)	5 (23.8)	6 (28.6)	
		Sometimes used	22 (50.0)	10 (22.7)	12 (27.3)	
		Often used	27 (75.0)	3 (8.3)	6 (16.7)	
		Always used	19 (76.0)	3 (12.0)	3 (12.0)	
	Official websites	Never used	29 (63.0)	10 (21.7)	7 (15.2)	
		Sometimes used	22 (50.0)	8 (18.2)	14 (31.8)	
		Often used	18 (75.0)	2 (8.3)	4 (16.7)	
		Always used	9 (75.0)	1 (8.3)	2 (16.7)	
	Scientific literature	Never used	44 (60.3)	16 (21.9)	13 (17.8)	
		Sometimes used	22 (61.1)	3 (8.3)	11 (30.6)	
		Often used	9 (64.3)	2 (14.3)	3 (21.4)	
		Always used	3 (100.0)	0 (0.0)	0 (0.0)	
	“If the vaccine is available I would take it.”	Yes	40 (78.4)	5 (9.8)	6 (11.8)	
		No	15 (37.5)	14 (35.0)	11 (27.5)	<0.001
		Don't know	23 (65.7)	2 (5.7)	10 (28.6)	
	“I believe that it is important to inform the public daily about the number of new cases of COVID-19 infection.”	Yes	49 (73.1)	4 (6.0)	14 (20.9)	
		No	22 (44.9)	16 (32.7)	11 (22.4)	
		Don't know	7 (70.0)	1 (10.0)	2 (20.0)	0.003
	“I believe that people suffering from the infection of COVID-19 are stigmatized.”	Yes	30 (69.8)	5 (11.6)	8 (18.6)	
		No	36 (57.1)	14 (22.2)	13 (20.6)	
		Don't know	12 (60.0)	2 (10.0)	6 (30.0)	
